# Updates on Diversity Among Cardiology-Related Fellowships

**DOI:** 10.7759/cureus.47111

**Published:** 2023-10-16

**Authors:** Casey T Walk, Rebekah Lantz

**Affiliations:** 1 Surgery, Wright State University Boonshoft School of Medicine, Dayton, USA; 2 Internal Medicine, Miami Valley Hospital, Dayton, USA

**Keywords:** underrepresented gropus, frieda, healthcare outcomes, gme, gender discrepancy, osteopath representation, diversity and equity in medicine, cardio thoracic surgery, cardiovascular fellowship, cardiology

## Abstract

Within the United States (US) medical system, diversity in healthcare is a growing concern although studies have shown improved patient outcomes when healthcare teams are diverse. We were interested in cardiology-related fellowships from internal medicine and surgical specialties to understand how females, osteopaths (DOs), and non-US graduates were represented compared to males, allopathic medical doctors (MD), and US-graduated peers. We obtained data about accredited cardiology fellowship programs from the Fellowship and Residency Electronic Interactive Database Access System (FRIEDA™) for 2022-2023 and determined statistical significance for male/female, DO/MD, and US/non-US graduate status by reviewing program sites. Statistical analysis utilized SAS Studio 3.8, version 9.4 (SAS Institute, Inc., Cary, NC) and Wilson score for confidence intervals. Cardiology-related fellowships from internal medicine and surgery backgrounds showed generalized marked disparities (p<0.001) with only a couple of exceptions. For Interventional Cardiology, non-US graduates were well represented (p=0.3775), and for Heart Failure & Transplant Cardiology, females were represented equally (p=0.0863). For all other specialties and values, females, DOs, and non-US graduates were underrepresented. Despite conversations about diversity, underrepresentation persists. We encourage further steps to address barriers preventing underrepresented groups from advancing to their full potential in leadership and careers. Increasing diversity promotes competence, empathy, communication, and inclusive patient care.

## Introduction

Cardiovascular disease continues to be the leading cause of morbidity and mortality worldwide for most patients in the United States (US) despite geography, race, ethnicity, and gender [1]. Despite this, disparities continue to be present in cardiology-related fields, across internal medicine and surgery backgrounds. Lack of diversity is termed Underrepresented in Medicine (UIM) by the Association of American Medical College (AAMC), which is defined as racial and ethnic populations who are underrepresented in the medical profession compared to the general population [2]. The topic has been studied from multiple perspectives within Graduate Medical Education (GME) [3-5]. 

When demographics are observed among cardiology-related specialties, disparities have been adequately described in the last decade [6,7]. Discussions have led to many changes and initiatives to increase UIM applicants and trainees, specific to race and gender [8-10]. However, less widely discussed is the difference in GME and fellowship training opportunities for doctors of osteopathic medicine (DOs) and non-US graduates within these fields [11,12].

The objective of this study was to evaluate outcomes of fellowship match details from the most recent matriculation year, 2022-2023. We observed the diversity of accepted applicants by male versus female gender, allopathic medical doctor (MD) versus DO degree, and US versus non-US medical education.

## Materials and methods

Information was obtained from the Fellowship and Residency Electronic Database Access system (FRIEDA™) for the matriculation year 2022-2023 [13]. Our search included internal medicine and surgical cardiology-associated fellowship training programs. Cardiology-related specialties as defined by FRIEDA™ that we included were Cardiovascular Disease, Interventional Cardiology, Clinical Cardiac Electrophysiology, and Advanced Heart Failure and Transplant Cardiology from an internal medicine discipline. Vascular Surgery and Thoracic Surgery (including Cardiothoracic Surgery) represented surgical disciplines.

**Figure 1:**
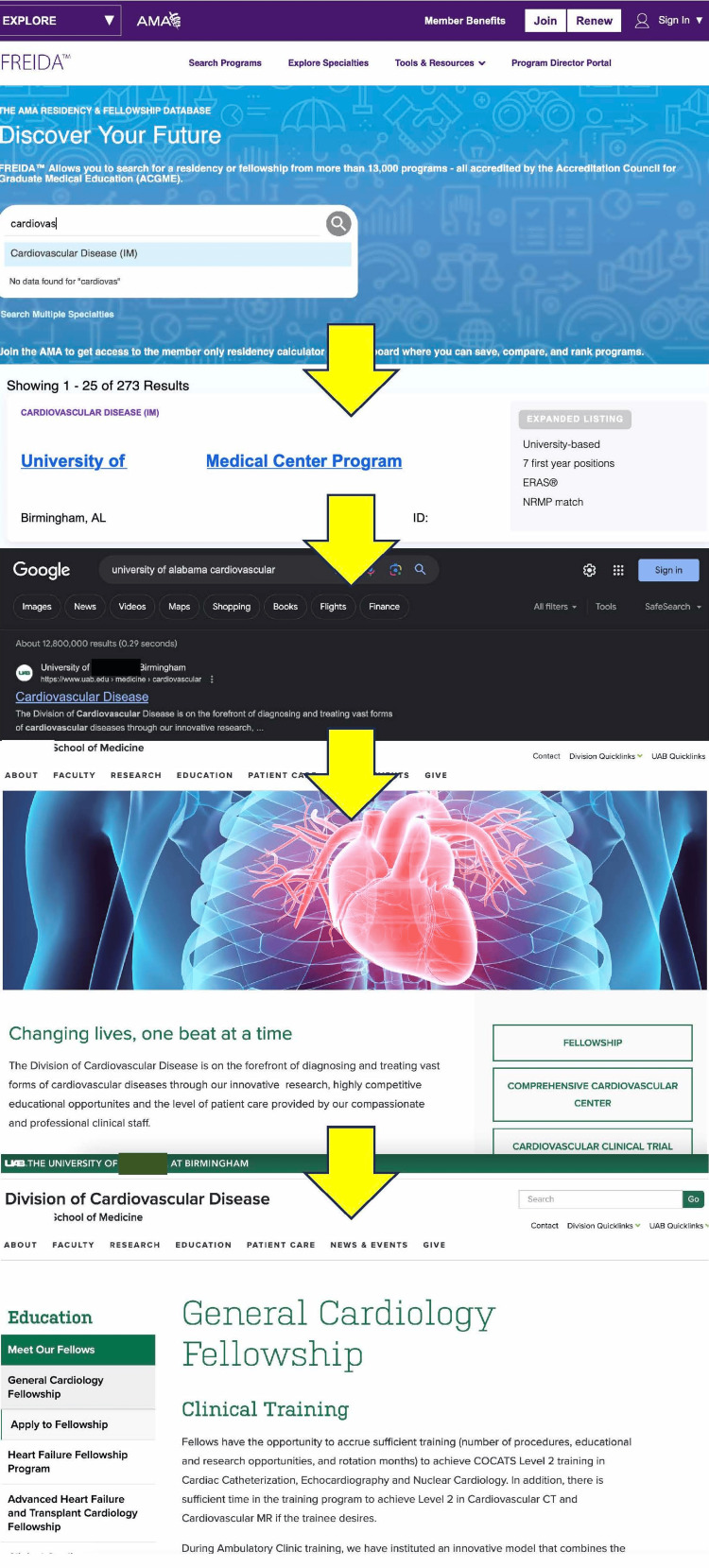
Flowsheet for initial program data retrieval

**Figure 2:**
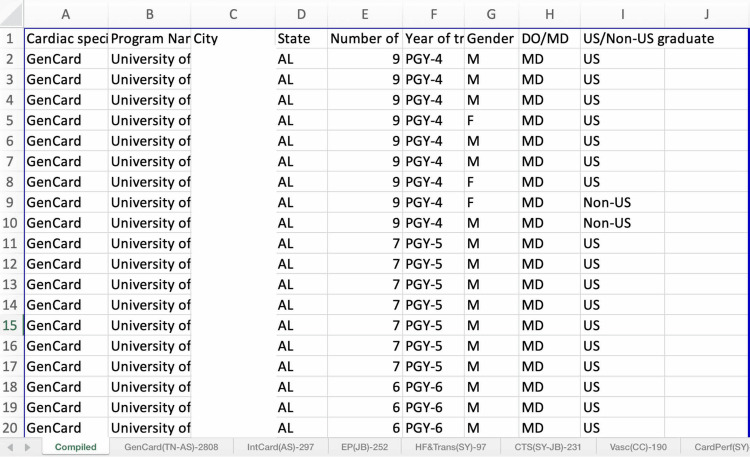
Excel sheet formatting for data collection

**Figure 3:**
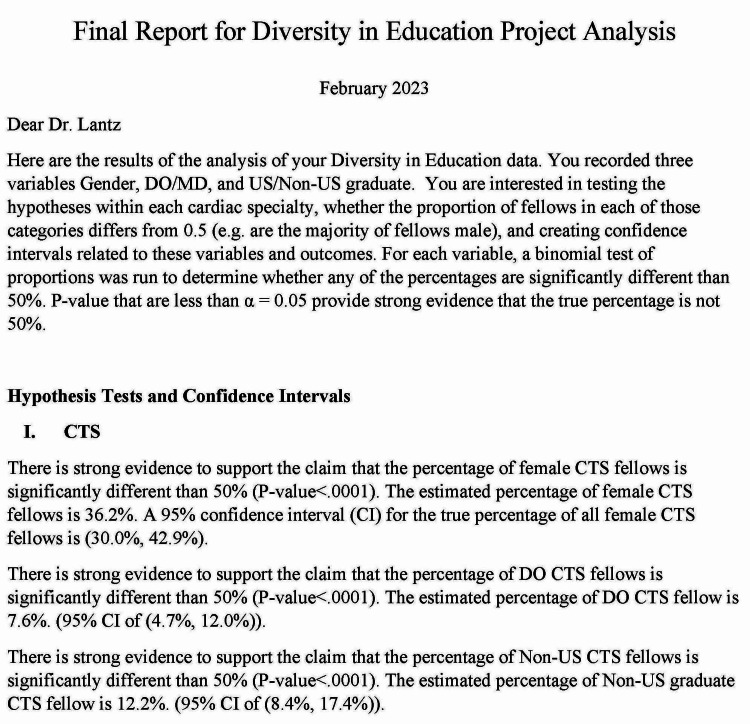
Statistician return letter with analysis on SAS Studio 3.8, version 9.4 (SAS Institute, Inc., Cary, NC)

Queried data included program name, region, and basic demographics: male/female, MD/DO, and US/non-US graduate (see Figure 1 in the Appendix). We could not obtain further information on another important demographic such as race or ethnicity as there would be a subjective component. We stated that we would not reach out to fellows so as not to involve human subject research. All of our data was available via online search engines, and collection was provided by student authors as noted in acknowledgments. The data then underwent statistical analysis with our research department and was returned to us as a data file. We were in consistent correspondence regarding the needs of our study (see Figures 2, 3 in the Appendix).

This study did not include newly accredited programs due to the limited information available. Paucity of fellow demographics would skew the data. For each variable, a binomial test of proportions determined significant differences from 50% (p<0.05). SAS Studio 3.8, version 9.4 (SAS Institute, Inc., Cary, NC) was used in the statistical analysis and Wilson score for confidence intervals [14].

## Results

Results from statistical analysis are shown in Table 1.

**Table 1:** Diversity across cardiology-related fellowships CI, confidence interval; DO, doctor of osteopathic medicine; US, United States.

Specialty	Frequency (%)	95% CI (%)	p-value
Internal medicine based			
General Cardiology			
Female gender	749 (27.3)	(26,29)	<0.0001
DO specialty	276 (10.1)	(9,11)	<0.0001
Non-US graduate	888 (32.5)	(31,34)	<0.0001
Interventional Cardiology			
Female gender	35 (16.2)	(12,22)	<0.0001
DO specialty	17 (7.8)	(5,12)	<0.0001
Non-US graduate	115 (53)	(46,60)	0.3775
Electrophysiology			
Female gender	29 (14.2)	(10,20)	<0.0001
DO specialty	16 (7.6)	(5,13)	<0.0001
Non-US graduate	75 (38.1)	(32,45)	<0.0001
Heart Failure & Transplant			
Female gender	12 (35.3)	(21,52)	0.0863
DO specialty	3 (8.3)	(3,22)	<0.0001
Non-US graduate	9 (27.3)	(15,44)	0.0090
Surgery based			
Cardiothoracic Surgery			
Female gender	76 (36.2)	(30,43)	<0.0001
DO specialty	16 (7.6)	(5,12)	<0.0001
Non-US graduate	25 (12.2)	(8,17)	<0.0001
Vascular Surgery			
Female gender	40 (32)	(24,41)	<0.0001
DO specialty	21 (16.2)	(11,23)	<0.0001
Non-US graduate	12 (13.3)	(8,22)	<0.0001

Internal medicine-based fellowships

Cardiovascular/General Cardiology fellowship showed demographic disparity for the three demographics. Female gender frequency was 749 (27.3%) (CI 26, 29) (p<0.0001), DO frequency 276 (10.1%) (CI 9,11) (p<0.0001), and non-US graduate frequency 888 (32.5%) (CI 31,34) (p<0.0001). Again, this suggests that females, DOs, and non-US graduates were underrepresented for Cardiovascular fellowship. This portion of data was previously published by Hughes et al. in Open Cardiology Research [15]. Regional data will be presented at CHEST 2023 in Hawaii in October.

Interventional Cardiology showed representation for non-US graduates only. Statistics were as follows with a female frequency of 35 (16.2%) (CI 12,22) (p<0.0001) and DO training with frequency 17(7.8%) (CI 5,12) (p<0.0001). Non-US frequency was 115 (53%) (CI 46,60), with p=0.3775. Females and DOs were underrepresented while non-US graduates appeared to vary from a 0.5 (50%) representation and a p>0.05 for Interventional Cardiology.

Electrophysiology (EP) fellowship showed demographic disparity across the three backgrounds with female gender frequency 29 (14.2%) (CI 10,20) (p<0.0001), DO frequency 16 (7.6%) (CI 5, 13) (p<0.0001), and non-US graduate frequency 75 (38.1%) (CI 32,45) (p<0.0001). Females, DOs, and non-US graduates were underrepresented for EP.

Heart Failure & Transplant from an internal medicine discipline showed osteopathic and international medical graduate (IMG) disparity with DO frequency 3 (8.3%) (CI 3,22) (p<0.0001) and non-US graduate frequency 9 (27.3%) (CI 15,44) (p<0.0001). However, there was not a significant difference in gender with a female frequency 12 (35.3%) (CI 21,52) where p=0.0863, indicating that females in Heart Failure & Transplant were at least equally represented. While the detail frequency 12 (35.3%) appears to fall <0.5 representation, our statisticians had adjusted for missing gender values for this topic of Heart Failure & Transplant. Given the small number of total candidates (N=34) in programs able to be assessed by available program information, gender fell outside the range of statistical significance and resulted in apparent equal female representation for all intents and purposes.

Surgery-based fellowships

Thoracic surgery, including Cardiothoracic surgery (CTS), showed demographic disparity with female frequency 76 (36.2%) (CI 30, 43) (p<0.0001), DO frequency 16 (7.6%) (CI 5,12) (p<0.0001), and non-US graduate frequency 25 (12.2%) (CI 8,17) (p<0.0001). This is notably significant for all demographic fields. That is to say that females, DOs, and non-US graduates were underrepresented for CTS.

Lastly, vascular surgery was significant across the three demographics with female gender frequency 40 (32%) (p<0.0001) (CI 24,31), DO frequency 21 (16.2%) (CI 11,23) (p<0.0001), and non-US graduate frequency 12 (13.3%) (CI 8,22) (p<0.0001). Again, this was an example where females, DOs, and non-US graduates remained underrepresented.

We found trends across surgical specialties to be male, MD-trained, and US-graduate status. Our data specific to surgical-related fields of cardiology was previously published in Journal of Cardiology and Current Research [16].

Composite fields

Cardiology-related fellowship programs, from both internal medicine and surgery backgrounds, displayed gender disparity with a statistically significant difference in female frequency with the exception of Heart Failure & Transplant 12 (35.3) (CI 21,52) (p=0.0863). All specialties demonstrated osteopathic disparity with lesser frequencies of DO-trained physicians. Finally, all fellowships displayed disparity for non-US graduates with the exception of Interventional Cardiology 115 (53) (CI 46,60) (p=0.3775), in which non-US graduates appeared to be represented more than US graduates. Results for these fields are shown at the beginning of this section in Table 1.

## Discussion

Cardiology-related specialties continue to lack diversity over the past decade despite multiple efforts toward change [17]. The AAMC termed this group UIM for racial, ethnic, and other groups that are underrepresented in medicine compared to the general population [2]. It is well known that underrepresentation has both subliminal and overt consequences on population health and the quality of care that patients receive [17-19]. Additionally, current representation may have implications for the career choice of trainees [3,17].

Our data within GME notes that females, DOs, and non-US graduates continued to be underrepresented in the recent 2022-2023 application cycle across cardiology-related fellowships with only a couple of exceptions. Heart Failure & Transplant represented females well (p=0.0863) and Interventional Cardiology represented non-US graduates (p=0.3775). DOs were consistently underrepresented across specialties, regardless of internal medicine or surgery backgrounds. If current trends do not actively improve, it is unlikely they will improve. In the general desire to belong, providers look at opportunities where seniors and staff look like them and will support them [3]. The same is true for patients who are more likely to expound on their clinical history with a trusted provider who may be female like them, prefer a holistic approach like them, or be non-US born like them in a diversifying population [3,17-19].

GME groups have observed the concern from multiple angles and levels of training [2-5,19]. The AAMC outlines strategies to make positive strides toward inclusion including identifying unique populations, using validated tools to measure engagement and retention, collaborating with diversity partners, endorsing support groups, participating in national heritage celebrations, implementing unconscious bias training to faculty and staff, facilitating mentorship, providing scholarships and grants, and meeting with representatives regularly [2]. Dye and Lantz suggest increasing outreach at an early educational level as well as advocating opportunities for leadership and research at early career stages. The focus should be on diverse groups [19]. Alongside objective goals in which numerical strides can be applied, it is essential to normalize a culture that does not allow discrimination and aggressions or microaggressions toward persons of different faith, skin color, socioeconomic background, gender, choice of training, or country of origin [19]. The purpose of diversity is not achieved without a supportive culture in tandem.

Several limitations of our study exist. Data was obtained from FRIEDA™ by multiple student co-authors for their assigned specialty. Variation was addressed with the oversight of the attending author who refined searches and did intermittent quality checks of the data turned in on a by-student basis. Fellowships were reviewed for male/female status, MD/DO, and US/non-US graduates. Gender status was reviewed by name and photos where applicable, which we expect contained minimal errors. Every program did not openly display fellow photo or information on the completed degree or country of origin. A degree other than MD/DO was automatically considered a non-US graduate. For direct missing fellow demographic information, professional websites such as LinkedIn and Doximity were reviewed. Still there was not information for every fellow for every program. Additionally, this study did not include newly accredited programs due to the paucity of fellow demographics that would skew the data. These may have contributed to further information if included. Further detailed information regarding our data fields can be provided upon request and replicated for future studies.

## Conclusions

Diversity and inclusion in cardiology-related fellowship programs are historically lacking, and our data from the recent matriculation year shows the same despite research and attention to the topic. The authors desire to add an urgency to change and improve representation for females, DOs, and non-US graduates with the intention of improving the important health outcomes of the populations we serve. The goal would be to match diversity in the recruitment and maintenance of healthcare providers with the trends of diversity in the general population.
